# Epidural morphine improves postoperative analgesia in patients after total knee arthroplasty: A randomized controlled trial

**DOI:** 10.1371/journal.pone.0219116

**Published:** 2019-07-01

**Authors:** Zhao-Ting Meng, Fan Cui, Xue-Ying Li, Dong-Xin Wang

**Affiliations:** 1 Department of Anesthesiology and Critical Care Medicine, Peking University First Hospital, Beijing, China; 2 Department of Biostatistics, Peking University First Hospital, Beijing, China; Public Library of Science, UNITED KINGDOM

## Abstract

**Background:**

Patients after total knee arthroplasty (TKA) often develop moderate to severe pain. This study compared the analgesic effect of low-dose epidural morphine vs. a comparable saline injection in patients following TKA surgery.

**Methods:**

This randomized, double-blinded, and placebo-controlled trial was conducted in a tertiary hospital in Beijing between July 1, 2017 and May 30, 2018. One hundred and ten patients following TKA under combined spinal-epidural anesthesia were randomized to receive either epidural morphine (2 mg diluted to 5 ml normal saline, the epidural morphine group) or placebo (5 ml normal saline, the placebo group). For all patients, single-injection femoral nerve block was performed, and a supplementary patient-controlled intravenous analgesia pump was provided. The severity of pain was assessed with the numerical rating scale (NRS, an 11-point scale where 0 = no pain and 10 = the worst pain) at 6, 12, 24, 36, and 48 hours after surgery. The primary endpoint was moderate to severe pain (NRS pain score ≥4) within 48 hours after surgery.

**Results:**

The percentage with moderate to severe pain within 48 hours was lower in the epidural morphine group than in the placebo group (58.2% [32/55] with epidural morphine vs. 76.4% [42/55] with placebo; OR 0.43, 95% CI 0.19–0.98; p = 0.042). Furthermore, the cumulative morphine consumption within 48 hours was lower (18.4±6.1 mg vs. 22.4±7.3 mg; p = 0.002) whereas the mental component summary score of 30-day quality of life was higher (63.8±2.9 vs. 61.9±4.2; p = 0.008) in the epidural morphine group than in the placebo group.

**Conclusions:**

For patients following TKA, the addition of epidural morphine to single-injection femoral nerve block improves the quality of analgesia within 48 hours, without increasing adverse events.

**Trial registration:**

ClinicalTrials.gov NCT03203967.

## Introduction

Total knee arthroplasty (TKA) is an important therapy for patients with later-stage knee osteoarthritis to relieve pain and improve quality of life. However, many patients suffer from moderate to severe postoperative pain that impede them from participating early physical therapy and recovering joint function. In addition, severe postoperative pain also contributes to immobility-related complications like deep vein thrombosis and delay hospital discharge [1–5]. Although the analgesic techniques have been greatly improved, the effect is far from optimal [6–8]. Femoral nerve block is commonly used for analgesia after open knee surgery [9]. But single-injection femoral nerve block alone is not satisfactory because of limited blocking range [10–13] and duration [14–16]; therefore, it is usually combined with supplemental analgesics which may produce opioid-associated side effects.

Low-dose epidural morphine is commonly used for analgesia after cesarean section. The effect of single dose morphine lasts more than 20 hours, with low incidences of itching, nausea, vomiting, and respiratory depression [17,18]. We hypothesized that, for patients undergoing TKA, combined use of low-dose epidural morphine and single-injection femoral nerve block could improve the effect of postoperative analgesia, reduce the consumption of intravenous opioids and decrease opioid-associated side effects. The purpose of this study was to compare the analgesic effect of low-dose epidural morphine vs. a comparable saline injection in patients following TKA.

## Method

### Study design

This was a randomized, double-blind, and placebo-controlled one-center trial with a two-arm parallel-group design to prove the superiority hypothesis. The study protocol (S1 Text) was approved by the Ethics Committees of Peking University First Hospital (2017[1308]) on April 12, 2017, and was registered with ClinicalTrials.gov (NCT03203967) on June 29, 2017 (last updated May 31, 2018). The authors confirm that all ongoing and related trials for this intervention were registered. The protocol was deposited in protocols.io, with a DOI link of http://dx.doi.org/10.17504/protocols.io. [dx.doi.org/10.17504/protocols.io.wanfade]. Written informed consent obtained from each patient.

### Participants

Potential participants were screened the day before surgery. The inclusion criteria were adult patients (age of 18 years or older) who were scheduled to undergo unilateral TKA under combined spinal-epidural anesthesia. Patients who met any of the following criteria were excluded: (1) age higher than 90 years; (2) presence of any contraindication to neuraxial anesthesia or peripheral nerve block; (3) use of opioid analgesics during the last month; (4) unable to understand Numeric Rating Scale for pain evaluation or existence of language barrier; (5) severe renal insufficiency (requirement of renal replacement therapy); (6) history of asthma; or (7) American Society of Anesthesiologists (ASA) classification of grade IV or higher.

After obtaining the written informed consents, investigators responsible for patient recruitment collected detailed baseline data including demographic characteristics, diagnosis, comorbidities, current medication, NYHA classification, American Society of Anesthesiologists (ASA) classification, history of previous surgery and anesthesia, as well as important laboratory test results.

### Randomization, intervention and anesthesia management

The random numbers were generated in a 1:1 ratio with a block size of 4 by an independent biostatistician using the SAS statistical package version 9.3 (SAS Institute, Cary, NC, USA). The results of randomization were sealed in sequentially numbered envelops and stored at the site of investigation until the end of the study.

During the study period, each enrolled patient was assigned a recruitment number. The study drugs, either 2 mg morphine in 5 mL normal saline or 5 mL normal saline, were prepared according to the randomization sequence by a study coordinator (JTT) who did not participate in the rest of the study. The prepared drugs were contained in 5 mL syringes with same appearance, labeled with the recruitment numbers, and provided to the anesthesiologists taking care of the enrolled patients. All patients, health-care team members, and investigators responsible for data collection and postoperative follow-up were masked from group allocation throughout the study period.

Combined spinal (with 0.5% hyperbaric bupivacaine) and epidural (with 2% lidocaine) anesthesia was performed for all patients. The dosage of local anesthetics (either spinal bupivacaine or epidural lidocaine) was determined by the anesthesiologists. Intraoperative sedation was provided with dexmedetomidine infusion (a loading dose of 0.4 μg/kg in 10 minutes, followed by a 0.2 μg/kg/h infusion) which was started once patients were hemodynamically stable after spinal anesthesia, and stopped before the end of surgery. The study drugs (morphine for patients in the epidural morphine group and normal saline for those in the placebo group) was administered at the end of surgery through the indwelling epidural catheter which was removed afterwards.

Patients were transferred to the post-anesthetic care unit (PACU), where single-injection femoral nerve block was performed with 20 mL of 0.5% ropivacaine under the guidance of ultrasonography and a nerve stimulator. A patient-controlled intravenous analgesia (PCIA) pump was also provided, which was established with 100 mL of 0.5 mg/mL morphine and programmed to deliver a 2 mL bolus with a lockout interval of 8–10 min and a background infusion of 0.5 mL/h. The PCIA pump was stopped at 48 hours after surgery. If the morphine solution in the pump was exhausted before that time, supplemental morphine of same concentration was provided to ensure a 48-hour postoperative patient-controlled analgesia.

### Outcome assessment and follow-up schedule

Intraoperative data including duration of anesthesia and surgery, name of surgery, types and doses of anesthetic drugs, and fluid balance were recorded. After surgery, patients were monitored in PACU for at least 30 minutes. Motor blockade of the lower limbs was estimated using a modified Bromage scale (0 = no motor block, able to lift extended limb off the bed; 1 = partial block, able to flex/extend the knee and ankle; 2 = partial block, only plantar flexion of the ankle possible; 3 = complete block, no voluntary movement of the limb) at PACU arrival and 30 minutes.

After surgery, two investigators (MZT and CF) visited patients at 6, 12, 24, 36, and 48 hours after surgery (1 hour earlier or later was allowed). The severity of pain at rest and with movement was assessed with the numerical rating scale (NRS, an 11-point scale where 0 = no pain and 10 = the worst pain). Motor blockade of the lower limbs was estimated using a modified Bromage scale. The numbers of required and given bolus injections by the PCIA pumps between the neighbouring time-points were counted. The occurrence of side effects (nausea, vomiting, pruritus, and dizziness), the uses and dosages of other analgesics, the volume of drainage, and the requirement of blood transfusion within 48 hours were recorded. The score of patients’ satisfactions (1 = poor, 2 = fair, 3 = good, 4 = excellent) was evaluated at 48 hours after surgery. Other postoperative date including time to ambulation, length of postoperative hospital stays, occurrence of complications within 30 days, and 30-day mortality after surgery were documented.

At 30 days after surgery, the quality of life was assessed with 12-item short-form (SF-12, it is summarized into physical and mental component summary scores, that range from 0 to 100, with higher scores indicating better quality of life); the severity of arthritic symptoms was assessed with WOMAC osteoarthritis index (score ranges from 0 to 96, with higher score indicating more severe symptoms). 30-day followed-up was performed by face-to-face interview, when patients came back to the hospital for a re-examination.

The primary endpoint was moderate to severe pain (NRS pain score ≥4) within 48 hours after surgery. Secondary endpoints included the cumulative morphine consumption and supplemental analgesics within 48 hours, the percentage with satisfied analgesia at 48 hours, time to ambulation, length of stay in hospital after surgery, the incidence of complications within 30 days after surgery, all cause 30-day mortality, as well as the quality of life (SF-12) and WOMAC Osteoarthritis Index at 30 days after surgery. Other predefined endpoints included the NRS pain scores (at rest and with movement), the percentage with moderate to severe pain and bolus of PCIA pump at various time-points after surgery.

### Statistical analysis

#### Sample size estimation

A study comparing continuous femoral nerve block vs. continuous femoral nerve block plus mini-dose spinal morphine for analgesia after TKA showed that, at 12 hours after surgery, the percentage with moderate to severe pain was 69.7% vs. 42.9%, respectively [19]. We assumed similar results in the present study. With significance and power set at 0.05 (two-sided) and 80%, respectively, the calculated sample size required to detect differences was 102 patients. Considering a drop-out rate of 10%, we planned to enroll 110 patients. Estimation of the necessary sample size for the chi-square test to compare two proportions was performed using normal approximation (Formula (9) in Julious 2010 [20]) with PASS 11.0 software (StataCorp. LP, College Station, TX).

#### Outcome analyses

All data were entered into database using the EpiData software package (EpiData 3.1, EpiData Association, Odense, Denmark) with a double-entry method. Continuous variables (cumulative morphine consumption within 48 hours, and scores of the SF-12 and WOMAC Osteoarthritis Index at 30 days) were analyzed using the unpaired t-test, with mean differences calculated with the covariance analysis. Ordinal data (kinds of supplemental analgesics within 48 hours) were analyzed with Mann-Whitney U test, with median differences calculated with the Hodges-Lehmann estimator. Categorical variables (percentage with moderate to severe pain, percentage with satisfied analgesia, incidence of complications within 30 days, and all-cause 30-day mortality) were analyzed using the Chi-squared test or Fisher exact test, with odds ratio (OR) calculated by logistic analysis. Time-to-event results (time to ambulation and length of stay in hospital after surgery) were analyzed using the Kaplan-Meier survival analysis, with the difference between groups tested by the log-rank test and hazard ratio (HR) calculated by Cox regression analysis. Data collected from the pain diaries (NRS pain scores at rest and with movement, moderate to severe pain at rest and with movement, and required and given bolus of PCIA pump at different time points) were analyzed using nonlinear mixed effects models. A two-sided P value of less than 0.05 was considered statistically significant. Outcome and safety data were analyzed in the intent-to-treat population. We also did per protocol analysis for the primary endpoint. No interim analysis was performed. The statistical analysis was performed with SPSS 19.0 statistical package (SPSS Inc, Chicago, Ill, USA) and STATA9.0 (Stata Corporation, College Station, Texas, USA).

## Results

### Patient population

**Fig 1:**
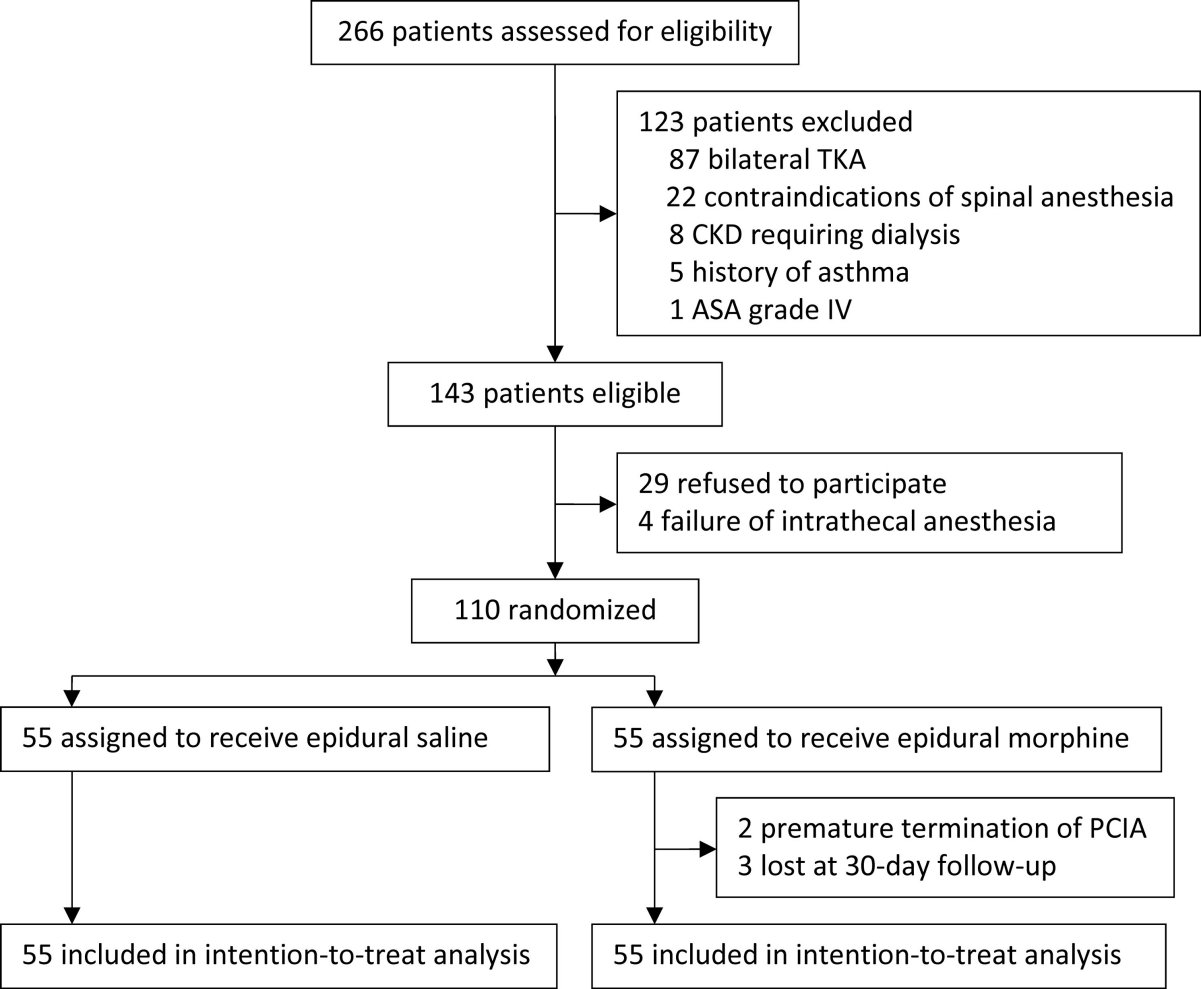
Flow diagram of the study. TKA, total knee arthroplasty; CKD, chronic kidney disease; ASA, American Society of Anesthesiologists; PCIA, patient-controlled intravenous analgesia.

From July 1, 2017 to May 30, 2018, 266 patients were screened for eligibility; 143 patients met the inclusion/exclusion criteria; 110 patients gave consents and were randomized into the study, among them 55 in the epidural morphine group and 55 in the placebo group. During the study period, 2 patients in the epidural morphine group discontinued the PCIA pump because of nausea and vomiting; 3 patients in the epidural morphine group were lost to follow up at postoperative day 30 (Fig 1). The last follow-up was performed on May 30, 2018. The baseline and intraoperative data were comparable between the two groups (Tables 1 and 2; S1 Dataset).

**Table 1:** Baseline data.

	Control group (n = 55)	Epidural morphine group (n = 55)
Age, year	69.3±7.3	67.5±7.3
Male gender	13 (23.6%)	11 (20.0%)
Body mass index, kg/m^2^	28.1±6.8	26.7±3.8
Comorbidities		
Hypertension	34 (61.8%)	31 (56.4%)
Coronary heart disease	11 (20.0%)	13 (23.6%)
Diabetes mellitus	9 (16.4%)	13 (23.6%)
Previous stroke	8 (14.5%)	7 (12.7%)
Arrhythmia	4 (7.3%)	1 (1.8%)
COPD	4 (7.3%)	1 (1.8%)
Chronic Smoking ^a^	4 (7.3%)	3 (5.5%)
NYHA function class		
II	54 (98.2%)	55 (100.0%)
III	1 (1.8%)	0 (0.0%)
ASA classification		
II	46 (83.6%)	50 (90.9%)
III	9 (16.4%)	5 (9.1%)

Results are presented as mean ± standard deviation or number (%). COPD, chronic obstructive pulmonary disease; NYHA, New York Heart Association; ASA, American Society of Anesthesiologists.

^a^ Smoking regularly for more than 1 year.

**Table 2:** Intraoperative data.

	Control group (n = 55)	Epidural morphine group (n = 55)	P value
Duration of anesthesia, min	149±21	153±28	0.372
Spinal bupivacaine, mg	12.5±1.5	12.5±1.0	0.815
Use of epidural lidocaine	3 (5.5%)	8 (14.5%)	0.112
Dexmedetomidine, μg	37±13	37±10	0.961
Duration of surgery, min	86±19	88±22	0.472
Total fluid infusion, ml	1478±298	1471±288	0.897
Estimated blood loss, ml	0 (0, 50)	0 (0, 100)	0.509

Results are presented as mean ± standard deviation, number (%) or median (interquartile range).

### Effectiveness analysis

The percentage of patients with moderate to severe pain (NRS pain score ≥4) within 48 hours was significantly lower in the epidural morphine group than in the placebo group (58.2% [32/55] with epidural morphine vs. 76.4% [42/55] with placebo; OR 0.43, 95% CI 0.19–0.98; P = 0.042) (Table 3). Per-protocol analysis also showed a similar difference in the percentage with moderate to severe pain between groups (58.5% [31/53] with epidural morphine vs. 76.4% [42/55] with placebo; OR 0.44, 95% CI 0.19–1.00; P = 0.047).

**Table 3:** Effectiveness outcomes.

	Control group (n = 55)	Epidural morphine group (n = 55)	Estimated difference (95% CI) ^a^	Value of the test statistic	Degrees of freedom	P value
**Primary outcome**						
Moderate to severe pain within 48 hrs	42 (76.4%)	32 (58.2%)	OR = 0.43 (0.19, 0.98)	4.129	1	0.042
**Secondary outcomes**						
Morphine consumption within 48 hrs, mg	22.4±7.3	18.4±6.1	Mean D = -4.02 (-6.54, -1.49)	-3.153	108	0.002
Kinds of supplemental analgesics within 48 hrs ^b^	2 (1, 2)	2 (1, 2)	Median D = 0.0 (0.0, 0.0)	1454.500	1	0.707
Satisfied with analgesia at 48 hrs ^c^	49 (89.1%)	52 (94.5%)	OR = 2.12 (0.50, 8.96)	1.079	1	0.489
Time to ambulation, ^d^	3.0 (2.8, 3.2)	2.0 (1.7, 2.3)	HR = 0.93 (0.64, 1.36)	0.278	1	0.598
Length of stay in hospital after surgery, ^d^	7.0 (6.8, 7.2)	7.0 (6.5, 7.5)	HR = 0.85 (0.58, 1.24)	1.040	1	0.308
Incidence of complications within 30 days	2 (3.6%)	0 (0.0%) (n = 52) ^d^	—	—	—	—
All cause 30-day mortality	0 (0.0%)	0 (0.0%) (n = 52)	—	—	—	—
Quality of life (SF-12) at 30 days ^e^						
Physical component summary, score	27.8±2.5	28.0±2.9 (n = 52)	Mean D = 0.23 (-0.80, 1.27)	0.447	105	0.656
Mental component summary, score	61.9±4.2	63.8±2.9 (n = 52)	Mean D = 1.91 (0.52, 3.30)	2.718	105	0.008
WOMAC Osteoarthritis Index at 30 days, score ^f^	26.2±4.9	26.4±3.3 (n = 52)	Mean D = 0.17 (-1.45, 1.78)	0.204	105	0.839

Results are presented as mean ± standard deviation, number (%), median (interquartile range) or median (95% CI). NRS, numerical rating scale; PCIA, patient-controlled intravenous analgesia.

^a^ Calculated as the epidural morphine group vs. or minus the placebo group.

^b^ Supplemental analgesics include flurbiprofen axetil (iv), parecoxib (iv), celecoxib (po), acetaminophen (po), tramadol (po) and/or oxycodone (po). All patients received one or more supplemental analgesics.

^c^ Patient’s satisfactions are divided into 4 grades, i.e., poor, fair, good, and excellent. Good and excellent are considered satisfactory.

^d^ 3 patients in the epidural morphine group were lost to follow up at postoperative day 30.

^e^ The SF-12 is summarized into physical and mental component summary scores, each ranges from 0 to 100, with higher score indicating better quality of life.

^f^ The score of WOMAC Osteoarthritis Index ranges from 0 to 96, with higher score indicating worse symptoms of arthritis.

**Fig 2:**
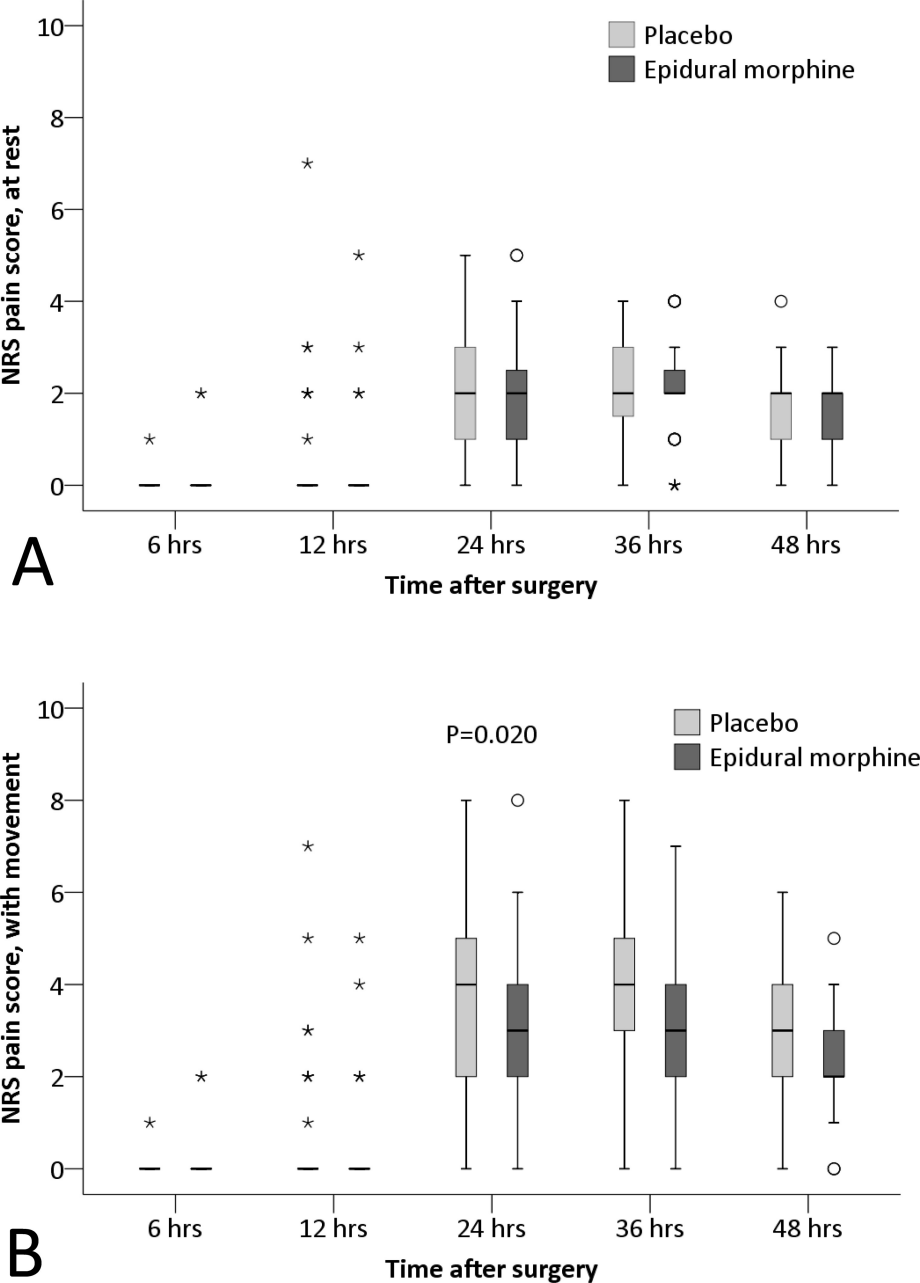
**The NRS pain scores at rest (A) and with movement (B).** The pain score with movement at different time-points were significantly lower in the epidural morphine group than in the placebo group (P = 0.020 [mixed-effects maximum likelihood regression model]). The box and whiskers plots show medians, interquartile ranges and outer ranges, and individual points mean mild outliers (o, which are outside 1.5 times of interquartile range) and extreme outliers (*, which are outside 3 times of interquartile range). NRS, numerical rating scale.

**Fig 3:**
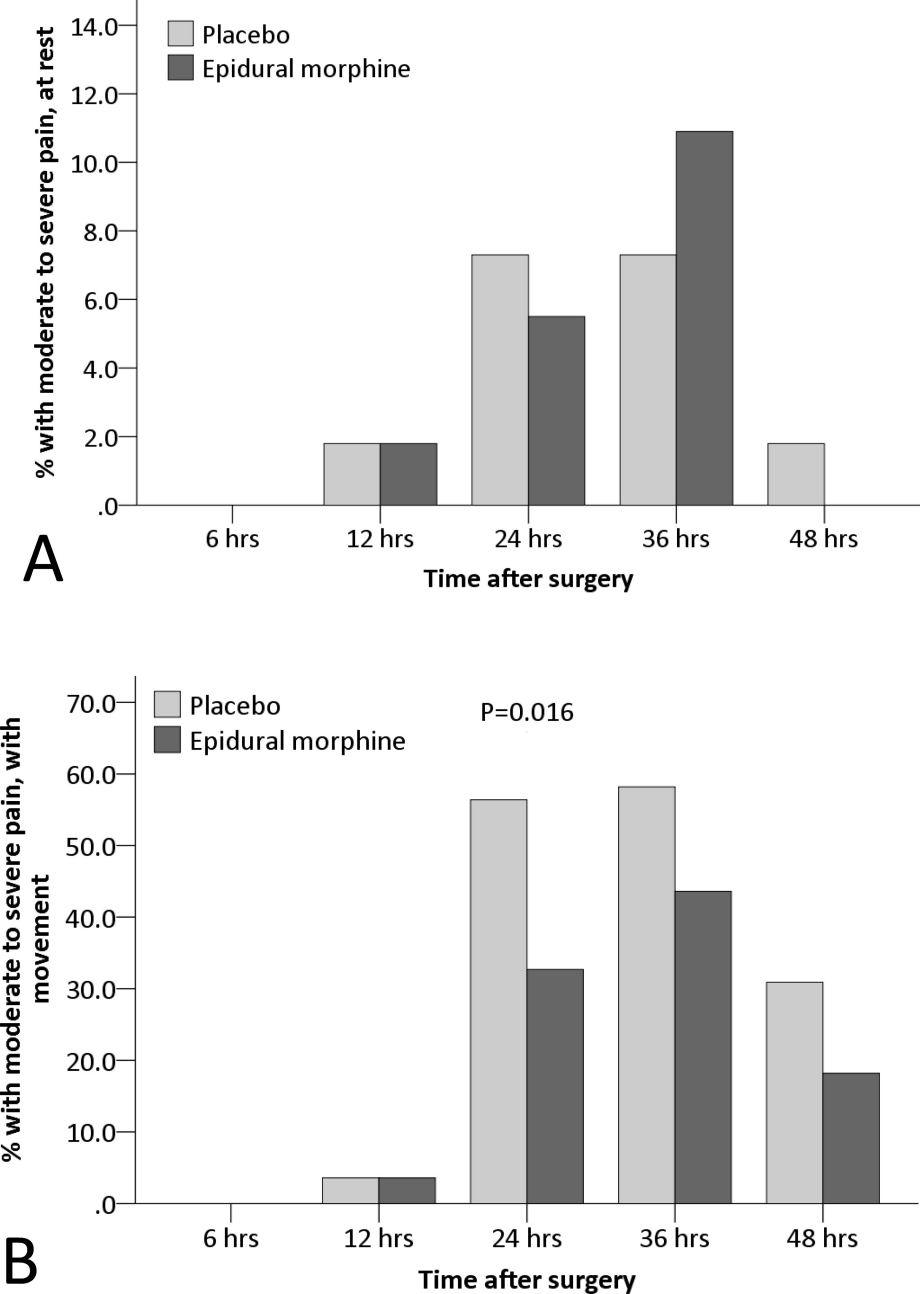
**The percentages with moderate to severe pain at rest (A) and with movement (B).** The percentage with movement at different time-points were significantly lower in the epidural morphine group than in the placebo group (P = 0.016 [mixed-effects logistic regression model]).

**Fig 4:**
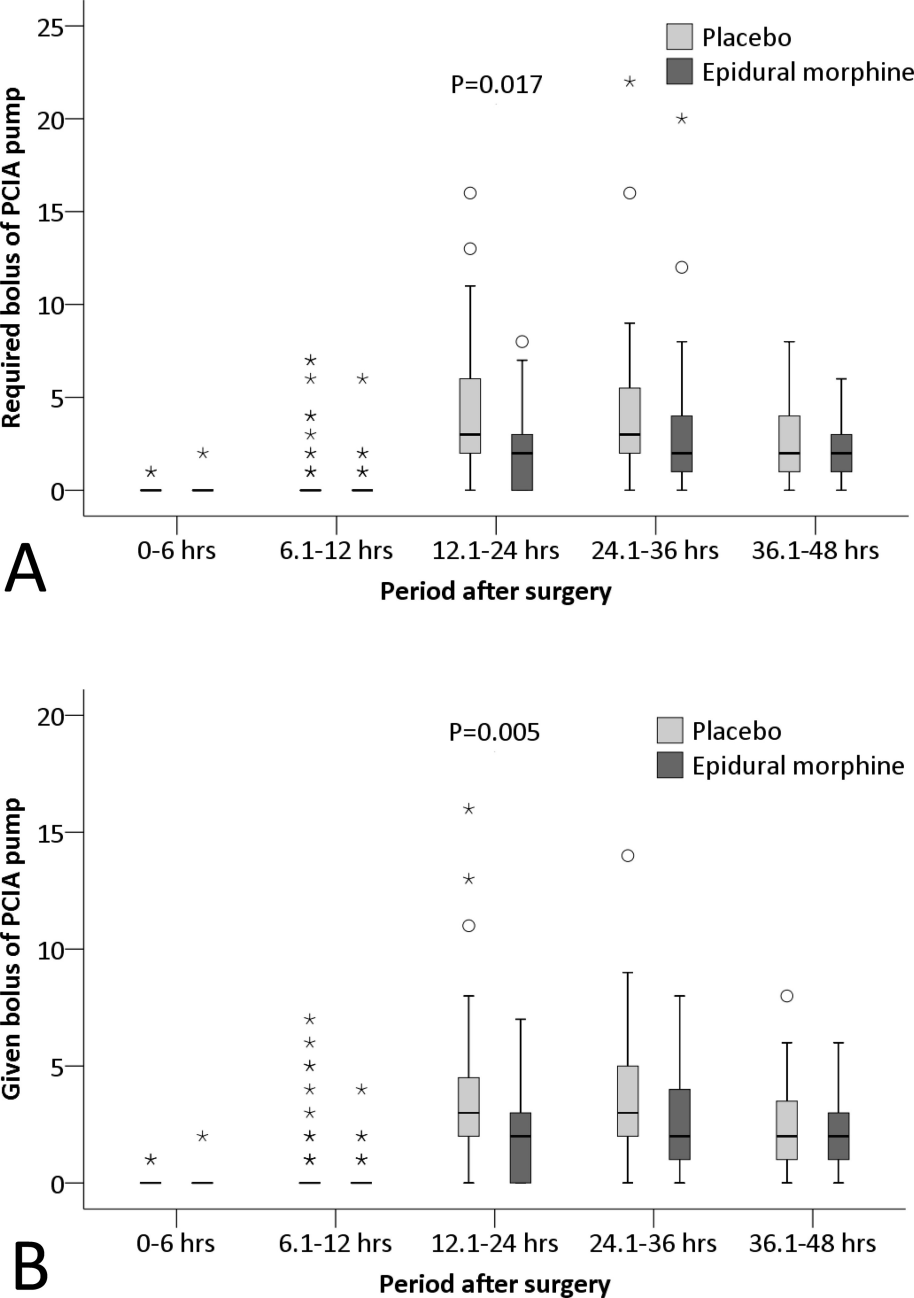
**The numbers of required (A) and given (B) boluses of the PCIA pump.** The numbers of both required and given boluses during different time-intervals were significantly less in the epidural morphine group than in the placebo group (P = 0.017 and 0.005, respectively [mixed-effects maximum likelihood regression model]). The box and whiskers plots show medians, interquartile ranges and outer ranges, and individual points mean mild outliers (o, which are outside 1.5 times of interquartile range) and extreme outliers (*, which are outside 3 times of interquartile range). PCIA, patient-controlled intravenous analgesia.

Furthermore, the cumulative morphine consumption within 48 hours was significantly lower (18.4±6.1 mg vs. 22.4±7.3 mg; P = 0.002), whereas the mental component summary score of quality of life (SF-12) at 30 days was significantly higher (63.8±2.9 vs. 61.9±4.2; P = 0.008) in the epidural morphine group than in the placebo group (Table 3). Among other predefined endpoints, the NRS pain score with movement and the percentage with moderate to severe pain with movement at different time-points were significantly lower (P = 0.020 [mixed-effects maximum likelihood regression model] and 0.016 [mixed-effects logistic regression model], respectively), and the numbers of required and given boluses by the PCIA pump during different time-intervals were significantly less (P = 0.017 and 0.005, respectively [mixed-effects maximum likelihood regression model]) in the epidural morphine group than in the placebo group (Figs 2, 3 and 4).

### Safety analysis

In all patients, motor blockage of lower limbs recovered completely within 6 hours after surgery. There were no significant differences between the two groups regarding the incidence of adverse events (Table 4).

**Table 4:** Safety outcomes.

		Control group (n = 55)	Epidural Morphine group (n = 55)	P value
Modified Bromage motor scale ^a^				
0 min	0	3 (5.5%)	7 (12.7%)	0.227
	1	13 (23.6%)	16 (29.1%)	
	2	22 (40.0%)	13 (23.6%)	
	3	17 (30.9%)	19 (34.5%)	
30 min	0	17 (30.9%)	22 (40.0%)	0.765
	1	18 (32.7%)	16 (29.1%)	
	2	13 (23.6%)	10 (18.2%)	
	3	7 (12.7%)	7 (12.7%)	
6 hrs	0	55 (100.0%)	55 (100.0%)	—
Nausea or vomiting within 48 hrs		16 (29.1%)	15 (27.3%)	0.832
Pruritus within 48 hrs		7 (12.7%)	7 (12.7%)	>0.999
Dizziness within 48 hrs		7 (12.7%)	10 (18.2%)	0.429

Results are presented as number (%).

^a^ Modified Bromage motor scale (0 = no motor block, able to lift extended limb off the bed; 1 = partial block, able to flex/extend the knee and ankle; 2 = partial block, only plantar flexion of the ankle possible; 3 = complete block, no voluntary movement of the limb).

## Discussion

Our results showed that, for patients following TKA, the addition of low-dose epidural morphine to single-injection femoral nerve block reduced the percentage with moderate to severe pain within 48 hours. It also decreased the cumulative morphine consumption within 48 hours and improved the mental health-related quality of life at 30 days. This intervention did not increase the incidence of side effects.

In the present study, TKA was performed under combined spinal-epidural anesthesia. Within 6 hours after surgery, no patient developed moderate to severe pain and bolus injection of PCIA pump was seldom required. These could be attributed to the residual effect of spinal anesthesia which was also confirmed by the existence of lower-limb motor blockage. From 6 to 12 hours after surgery, only a few patients had moderate to severe pain and bolus injections of PCA pump was occasionally required. One reason was that patients seldom move the surgical leg and, therefore, pain with movement was not induced; another reason was that the effect of single femoral nerve block and epidural morphine were satisfactory during this period. Studies showed that the mean duration of a single femoral nerve block (with 20 ml of 0.5% ropivacaine) lasts 799±364 minutes [21]. From 12 to 24 hours after surgery, the percentage with moderate to severe pain increased significantly and more bolus injection of PCIA pump was required. This was because the effect of spinal anesthesia disappeared, the effect of single femoral nerve block diminished, whereas the start of rehabilitative exercise induced more pain. Furthermore, the femoral nerve block does not affect the popliteal area [13]. However, the analgesic effect was significantly better in the epidural morphine group than in the placebo group, as manifested by a reduced median NRS pain score with movement by 1 point (from 4 [interquartile range 2, 5] to 3 [2, 4], Fig 2B) and a reduced percentage with moderate to severe pain with movement by 23.7% (from 56.4% to 32.7%, Fig 3B) at 24 hours after surgery. According to the results of Salaffi et al.,[22] a reduction of 1 point or more in the NRS pain score represented a minimal clinically important changes for the patient. This was because the low-dose epidural morphine was still working during that period, thus relieving pain more effectively in the epidural morphine group. Studies showed that the duration of a single low-dose epidural morphine may last for more than 20 hours [17,18]. From 24 to 36 hours after surgery, more patients suffered from moderate to severe pain than before and bolus injections were required frequently. This was mainly attributed to the absent effect of femoral nerve block and the decreasing effect of epidural morphine analgesia. The analgesic effect did not differ significantly between the two groups but was slightly better in the epidural morphine group. From 36 to 48 hours after surgery, the severity of pain began to decrease, similar trend also occurred in the percentage with moderate to severe pain and the number of acquired bolus injections. The effect of epidural morphine was significantly diminished during this period. There was no statistical significance in the analgesic effect between the two groups.

In the placebo group of the present study, the effect of spinal anesthesia completely disappeared at 12 hours after surgery; even though single femoral nerve block combined with intravenous PCA pump were used for postoperative analgesia, up to 76.4% of patients suffered from moderate to severe pain within 48 hours. Additional sciatic nerve block can improve postoperative analgesia by covering the popliteal area, but means more intervention, time and costs [23,24]. Additional spinal morphine also improves analgesic effect but increases the incidence of postoperative nausea and vomiting [13]. Continuous femoral nerve block provides long-lasting analgesic effect but has a relatively high rate of failure because of the dislodgement of the indwelling catheter [13,25]. In contrast, epidural administration of low-dose (2 mg) morphine provides an effective, safe, and easy-to-use method for postoperative analgesia [13,21]. Results of the present study showed that combination with low-dose epidural morphine improved postoperative analgesia and reduced opioid consumption within 48 hours, without increasing adverse events. Furthermore, our study revealed that low-dose epidural morphine significantly improved the mental health-related quality of life at 30 days after surgery, possibly due to improved analgesia and, thus, rehabilitation of the surgical limbs. Large sample size trials are needed to explore the effects of low-dose epidural morphine. However, although epidural morphine improved postoperative analgesia within 48 hours, the percentage of patients with moderate to severe pain remained high. Further improvements in the analgesic methods for patients following TKA surgery are required.

There were several limitations in our study. The update of the information in the trial registry does not affect the initial planning, but it should be noted that the sample size calculation is based on an approximation, which does not hold in small samples. The calculation results in 53 patients per group using nQuery Advisor for the Chi-Square test and 60 per group using Fisher exact test. Further taking our planned sample size of 102 with our initially planned 10% dropout rate, it results a corrected sample size of 113. Thus, the trial is slightly underpowered. Secondly, as a single-center study, the generalizability of our results may be limited. Thirdly, the difference of percentage with moderate to severe pain between the two groups used to calculate the sample size was 26.8%, whereas the actual difference in our results was 18.2%. This might have decreased the power of our study to detect the difference between groups. However, we do find that low-dose epidural morphine improved postoperative analgesia (S1 Checklist).

## Conclusions

For patients undergoing TKA, the addition of low-dose epidural morphine to single femoral nerve block reduces the percentage with moderate to severe pain within 48 hours; it also lowers the consumption of intravenous opioids within 48 hours and improves the mental health-related quality of life at 30 days after surgery, without increasing adverse events.

## Supporting information

S1 Checklist CONSORT checklist. (DOCX)

S1 Text Study protocol. (DOCX)

S1 Dataset Relevant data underlying the main results. (XLSX)
