# Supplementary material for: Epidural morphine improves postoperative analgesia in patients after total knee arthroplasty: A randomized controlled trial
Source: PLoS One. 2019 Jul 1;14(7):e0219116. doi: 10.1371/journal.pone.0219116 (PMC6602200; doi:10.1371/journal.pone.0219116)
Supplement: S1 Text — (DOCX) [file pone.0219116.s002.docx]

**Epidural morphine combined with single-injection femoral nerve block for postoperative analgesia in patients after total knee arthroplasty: a randomized controlled trial**

**Study protocol**

Department of Anesthesiology and Critical Care Medicine

Peking University First Hospital

Dong-Xin Wang, MD, PhD

Zhao-Ting Meng, MD

Version 1.0 (February 26, 2017)

**Objective**

The purpose of this study is to investigate the analgesic effect of low-dose epidural morphine added to single-injection femoral nerve block in patients following total knee arthroplasty (TKA) surgery.

**Background**

Total knee arthroplasty is an important therapy for patients with later-stage knee osteoarthritis to relieve pain and improve quality of life. However, many patients suffer from moderate to severe postoperative pain that impede them from participating early physical therapy and recovering joint function. In addition, severe postoperative pain also contributes to immobility-related complications like deep vein thrombosis and delay hospital discharge [1-5]. Although the analgesic techniques have been greatly improved, the effect is far from optimal [6-8]. Femoral nerve block is commonly used for analgesia after open knee surgery [9]. But femoral nerve block alone is not satisfactory because of limited blocking range [10-13] and duration [14-16]; therefore, it is usually combined with supplemental analgesics which may produce opioid-associated side effects.

Low-dose epidural morphine is commonly used for analgesia after cesarean section. The effect of single dose morphine lasts more than 20 hours, with low incidences of itching, nausea, vomiting, and respiratory depression [17,18]. We hypothesized that, for patients undergoing TKA, combined use of low-dose epidural morphine and single femoral nerve block could improve the effect of postoperative analgesia, reduce the consumption of intravenous opioids and decrease opioid-associated side effects. The purpose of this study is to compare the analgesic effect of low-dose epidural morphine combined with single-injection femoral nerve block and single-injection femoral nerve block alone in patients following TKA.

**Research protocol**

**Participants**

**Inclusion criteria:** Adult patients (age of 18 years or older) who are scheduled to undergo unilateral TKA under combined spinal-epidural anesthesia.

**Exclusion criteria:**

1. age higher than 90 years;
2. presence of any contraindication to neuraxial anesthesia or peripheral nerve block;
3. use of opioid analgesics during the last month;
4. unable to understand Numeric Rating Scale for pain evaluation or existence of language barrier;
5. severe renal insufficiency (requirement of renal replacement therapy);
6. history of asthma;
7. American Society of Anesthesiologists (ASA) classification of grade IV or higher.

**Sample size estimation**

A study comparing continuous femoral nerve block vs. continuous femoral nerve block with mini-dose spinal morphine for analgesia after TKA showed that, at 12 hours after surgery, the percentage with moderate to severe pain was 69.7% vs. 42.9%, respectively [19]. We assumed similar results in the present study. With significance and power set at 0.05 (two-sided) and 80%, respectively, the calculated sample size required to detect differences was 100 patients. Considering a drop-out rate of about 10%, we planned to enroll 110 patients. Sample size calculation was performed with the PASS 11.0 software (StataCorp. LP, College Station, TX).

**Enrollment**

Patients will be screened the day before surgery. And eligible patients will be enrolled for their consent in advance and written informed consent will be signed.

**Randomization, intervention and anesthesia management**

This is a randomized, double-blind, and placebo-controlled one-center trial. The random numbers were generated in a 1:1 ratio with a block size of 4 by an independent biostatistician using the SAS statistical package version 9.3 (SAS Institute, Cary, NC, USA). Each enrolled patient will be assigned a number according to the sequence of recruitment. The study drugs, either 2 mg morphine in 5 mL normal saline or 5 mL normal saline, will be prepared according to the randomization results by a study coordinator who will not participate in the rest of the study. The prepared drugs will be contained in 5 mL syringes with same appearance, labeled with the number of recruitments, and provided to the anesthesiologists taking care of the enrolled patients. The results of randomization and the preparation of study drugs will be recorded and sealed in sequentially numbered letters and stored at the site of investigation until the end of the study.

Combined spinal (with 0.5% hyperbaric bupivacaine) and epidural (with 2% lidocaine) anesthesia will be performed for all patients. The dosage of local anesthetics (either spinal bupivacaine or epidural lidocaine) will be determined by the anesthesiologists. Intraoperative sedation will be provided with dexmedetomidine infusion (a loading dose of 0.4 μg/kg in 10 minutes, followed by a 0.2 μg/kg/h infusion) which will be started once patients become hemodynamically stable after spinal anesthesia, and stopped before the end of surgery. The study drugs (morphine for patients in the epidural morphine [EDMO] group and normal saline for those in the control [CTRL] group) will be administered at the end of surgery through the indwelling epidural catheter which will be removed afterwards.

Patients will be transferred to the post-anesthetic care unit (PACU), where single-injection femoral nerve block will be performed with 20 mL of 0.5% ropivacaine under the guidance of ultrasonography and a nerve stimulator. A patient-controlled intravenous analgesia (PCIA) pump will also be provided, which is established with 100 mL of 0.5 mg/mL morphine and programmed to deliver a 2 mL bolus with a lockout interval of 8–10 min and a background infusion of 0.5 mL/h. The PCIA pump will be stopped at 48 hours after surgery. If the morphine solution in the pump is exhausted before that time, supplemental morphine of same concentration will be provided to ensure a 48-hour postoperative patient-controlled analgesia.

**Outcome assessment and follow-up schedule**

After obtaining the written informed consents, detailed baseline data including demographic characteristics, diagnosis, comorbidities, current medication, NYHA classification, American Society of Anesthesiologists (ASA) classification, history of previous surgery and anesthesia, as well as important laboratory test results will be collected.

Intraoperative data including duration of anesthesia and surgery, name of surgery, types and doses of anesthetic drugs, and fluid balance will be recorded. After surgery, patients will be monitored in PACU for at least 30 minutes. Motor blockade of the lower limbs will be estimated using a modified Bromage scale (0 = no motor block, able to lift extended limb off the bed; 1 = partial block, able to flex/extend the knee and ankle; 2 = partial block, only plantar flexion of the ankle possible; 3 = complete block, no voluntary movement of the limb) at PACU arrival and 30 minutes.

After surgery, investigators will visit patients at 6, 12, 24, 36, and 48 hours after surgery (1 hour earlier or later is allowed). The severity of pain at rest and with movement will be assessed with the numerical rating scale (NRS, an 11-point scale where 0 = no pain and 10 = the most severe pain). Motor blockade of the lower limbs will be estimated using a modified Bromage scale. The numbers of required and given bolus injections by the PCIA pumps between the neighbouring time-points will be counted. The occurrence of side effects (nausea, vomiting, pruritus, and dizziness), the uses and dosages of other analgesics, the volume of drainage, and the requirement of blood transfusion within 48 hours will be recorded. The score of patients’ satisfactions (1 = poor, 2 = fair, 3 = good, 4 = excellent) will be evaluated at 48 hours after surgery. Other postoperative date including time to ambulation, length of postoperative hospital stays, occurrence of complications within 30 days, and 30-day mortality after surgery will be documented.

At 30 days after surgery, the quality of life will be assessed with 12-item short-form; the severity of arthritic symptoms will be assessed with WOMAC osteoarthritis index. 30-day followed-up will be performed by face-to-face interview, when patients come back to the hospital for a re-examination.

**Endpoints**

The primary endpoint is the percentage of patients with moderate to severe pain (NRS ≥ 4) within 48 hours after surgery.

Secondary endpoints include the cumulative morphine consumption and supplemental analgesics within 48 hours, the percentage with satisfied analgesia at 48 hours, time to ambulation, length of stay in hospital after surgery, the incidence of complications within 30 days after surgery, all cause 30-day mortality, as well as the SF-12 Physical Component Summary (PCS) and Mental Component Summary (MCS) scores and WOMAC Osteoarthritis Index at 30 days after surgery.

Other predefined endpoints include the NRS pain scores (at rest and with movement) and the percentage with moderate to severe pain at various time-points after surgery.

**Statistical analysis**

All data will be entered into database using the EpiData software package (EpiData 3.1, EpiData Association, Odense, Denmark) with a double-entry method. Continuous variables with normal distribution will be analyzed using the unpaired t-test. Mean differences will be calculated with the covariance analysis. Continuous variables with non-normal distribution or ordinal data will be analyzed with Mann-Whitney U test. Median differences will be calculated with the Hodges-Lehmann estimator. Categorical variables will be analyzed using the Chi-squared test or Fisher exact test, with odds ratio (OR) calculated by logistic analysis. Time-to-event results will be analyzed using the Kaplan-Meier survival analysis, with the difference between groups tested by the log-rank test and hazard ratio (HR) calculated by Cox regression analysis. Statistical analysis will be performed with SPSS 19.0 statistical package (SPSS Inc, Chicago, Ill, USA), with two-sided P value of less than 0.05 as statistical significance.

**Ethical issues**

When obtaining informed consent, investigators should give participants a detailed description of the purpose, methodology, potential benefits and risks of the study, and what kind of collaboration they need. Meanwhile investigators should tell the participants that they are completely voluntary to participate in this clinical study and can withdraw at any time during the trial. Refusing or withdrawing from this clinical trial will not affect the treatment and reasonable medical rights of the participants.

**Estimated study period**

From July 1, 2017 to May 30, 2018.

**References**

1. Capdevila X, Barthelet Y, Biboulet P, Ryckwaert Y, Rubenovitch J, [d'Athis](https://www-ncbi-nlm-nih-gov.proxygsu-uga1.galileo.usg.edu/pubmed/?term=d'Athis%20F%5bAuthor%5d&cauthor=true&cauthor_uid=10422923) F. Effects of perioperative analgesic technique on the surgical outcome and duration of rehabilitation after major knee surgery. Anesthesiology. 1999; 91(1):8–15. PMID:10422923
2. Singelyn FJ, Deyaert M, Joris D, [Pendeville E](https://www-ncbi-nlm-nih-gov.proxygsu-uga1.galileo.usg.edu/pubmed/?term=Pendeville%20E%5bAuthor%5d&cauthor=true&cauthor_uid=9661552), [Gouverneur JM](https://www-ncbi-nlm-nih-gov.proxygsu-uga1.galileo.usg.edu/pubmed/?term=Gouverneur%20JM%5bAuthor%5d&cauthor=true&cauthor_uid=9661552). Effects of intravenous patient-controlled analgesia with morphine, continuous epidural analgesia, and continuous three-in-one block on postoperative pain and knee rehabilitation after unilateral total knee arthroplasty. Anesth Analg. 1998; 87(1):88–92. PMID:9661552
3. Wang H, Boctor B, Verner J. The effect of single-injection femoral nerve block on rehabilitation and length of hospital stay after total knee replacement. Reg Anesth Pain Med. 2002; 27(2):139–44. PMID:11915059
4. Bonica J. Postoperative pain. In: Bonica J, editor. The Management of Pain. 2nd ed. Philadelphia: Lea & Febiger; 1990. pp. 461–80.
5. Pang WW, Hsu TC, Tung CC, [Hung CP](https://www-ncbi-nlm-nih-gov.proxygsu-uga1.galileo.usg.edu/pubmed/?term=Hung%20CP%5bAuthor%5d&cauthor=true&cauthor_uid=11125689), [Chang DP](https://www-ncbi-nlm-nih-gov.proxygsu-uga1.galileo.usg.edu/pubmed/?term=Chang%20DP%5bAuthor%5d&cauthor=true&cauthor_uid=11125689), [Huang MH](https://www-ncbi-nlm-nih-gov.proxygsu-uga1.galileo.usg.edu/pubmed/?term=Huang%20MH%5bAuthor%5d&cauthor=true&cauthor_uid=11125689). Is total knee replacement more painful than total hip replacement? Acta Anaesthesiol Sin. 2000; 38(3):143–8. PMID:11125689
6. Lamplot JD, Wagner ER, Manning DW. Multimodal pain management in total knee Arthroplasty: a prospective randomized controlled trial. J Arthroplast. 2013; 29(2):329–34. doi: [10.1016/j.arth.2013.06.005](https://doi.org/10.1016/j.arth.2013.06.005) PMID:23850410
7. Dong CC, Dong SL, He FC. Comparison of adductor canal block and femoral nerve block for postoperative pain in total knee arthroplasty: a systematic review and meta-analysis. Medicine (Baltimore). 2016; 95(12): e2983. doi: 10.1097/MD.0000000000002983 PMID:27015172
8. [Stevenson KL](https://www-ncbi-nlm-nih-gov.proxygsu-uga1.galileo.usg.edu/pubmed/?term=Stevenson%20KL%5bAuthor%5d&cauthor=true&cauthor_uid=29628682), [Neuwirth AL](https://www-ncbi-nlm-nih-gov.proxygsu-uga1.galileo.usg.edu/pubmed/?term=Neuwirth%20AL%5bAuthor%5d&cauthor=true&cauthor_uid=29628682), [Sheth N](https://www-ncbi-nlm-nih-gov.proxygsu-uga1.galileo.usg.edu/pubmed/?term=Sheth%20N%5bAuthor%5d&cauthor=true&cauthor_uid=29628682). Perioperative pain management following total joint arthroplasty: A review and update to an institutional pain protocol. [J Clin Orthop Trauma.](https://www-ncbi-nlm-nih-gov.proxygsu-uga1.galileo.usg.edu/pubmed/29628682) 2018; 9(1):40-45. doi: 10.1016/j.jcot.2017.09.014 PMID: 29628682
9. Seet E, Leong WL, Yeo AS, [Fook-Chong S](https://www-ncbi-nlm-nih-gov.proxygsu-uga1.galileo.usg.edu/pubmed/?term=Fook-Chong%20S%5bAuthor%5d&cauthor=true&cauthor_uid=16494145). Effectiveness of 3-in-1 continuous femoral block of differing concentrations compared to patient controlled intravenous morphine for post total knee arthroplasty analgesia and knee rehabilitation. Anaesth Intensive Care. 2006; 34(1):25-30. PMID:16494145
10. Tierney E, Lewis G, Hurtig JB, [Johnson D](https://www-ncbi-nlm-nih-gov.proxygsu-uga1.galileo.usg.edu/pubmed/?term=Johnson%20D%5bAuthor%5d&cauthor=true&cauthor_uid=3664912). Femoral nerve block with bupivacaine 0.25% for postoperative analgesia after open knee surgery. Can J Anaesth. 1987;34(5):455–8. doi: [10.1007/BF03014348](https://doi-org.proxygsu-uga1.galileo.usg.edu/10.1007/BF03014348) PMID:3664912
11. Weber A, Fournier R, Van Gessel E, [Gamulin Z](https://www-ncbi-nlm-nih-gov.proxygsu-uga1.galileo.usg.edu/pubmed/?term=Gamulin%20Z%5bAuthor%5d&cauthor=true&cauthor_uid=12442936). Sciatic nerve block and the improvement of femoral nerve block analgesia after total knee replacement. Eur J Anaesthesiol. 2002;19(11):834–6. PMID:12442936
12. Mansour NY, Bennetts FE. An observational study of combined continuous lumbar plexus and single-shot sciatic nerve blocks for post-knee surgery analgesia. Reg Anesth. 1996;21(4):287–91. PMID:8837184
13. Sundarathiti P, Ruananukul N, Channum T, [Kitkunasathean C](https://www-ncbi-nlm-nih-gov.proxygsu-uga1.galileo.usg.edu/pubmed/?term=Kitkunasathean%20C%5bAuthor%5d&cauthor=true&cauthor_uid=19301724), [Mantay A](https://www-ncbi-nlm-nih-gov.proxygsu-uga1.galileo.usg.edu/pubmed/?term=Mantay%20A%5bAuthor%5d&cauthor=true&cauthor_uid=19301724), [Thammasakulsiri J](https://www-ncbi-nlm-nih-gov.proxygsu-uga1.galileo.usg.edu/pubmed/?term=Thammasakulsiri%20J%5bAuthor%5d&cauthor=true&cauthor_uid=19301724), [Sodsee W](https://www-ncbi-nlm-nih-gov.proxygsu-uga1.galileo.usg.edu/pubmed/?term=Sodsee%20W%5bAuthor%5d&cauthor=true&cauthor_uid=19301724). A comparison of continuous femoral nerve block (CFNB) and continuous epidural infusion (CEI) in postoperative analgesia and knee rehabilitation after total knee arthroplasty (TKA). J Med Assoc Thai. 2009;92(3):328–34. PMID:19301724
14. Duarte VM, Fallis WM, Slonowsky D, [Kwarteng K](https://www-ncbi-nlm-nih-gov.proxygsu-uga1.galileo.usg.edu/pubmed/?term=Kwarteng%20K%5bAuthor%5d&cauthor=true&cauthor_uid=17027440), [Yeung CK](https://www-ncbi-nlm-nih-gov.proxygsu-uga1.galileo.usg.edu/pubmed/?term=Yeung%20CK%5bAuthor%5d&cauthor=true&cauthor_uid=17027440). Effectiveness of femoral nerve blockade for pain control after total knee arthroplasty. J Perianesth Nurs. 2006; 21(5):311–6. doi: [10.1016/j.jopan.2006.05.011](https://doi-org.proxygsu-uga1.galileo.usg.edu/10.1016/j.jopan.2006.05.011) PMID:17027440
15. Salinas FV, Liu SS, Mulroy MF. The effect of single-injection femoral nerve block versus continuous femoral nerve block after total knee arthroplasty on hospital length of stay and long-term functional recovery within an established clinical pathway. Anesth Analg. 2006;102(4):1234–9. doi:[10.1213/01.ane.0000198675.20279.81](https://doi-org.proxygsu-uga1.galileo.usg.edu/10.1213/01.ane.0000198675.20279.81) PMID:16551930
16. Soto Mesa D, Del Valle Ruiz V, Fayad Fayad M, [Cosío Carreño F](https://www-ncbi-nlm-nih-gov.proxygsu-uga1.galileo.usg.edu/pubmed/?term=Cos%C3%ADo%20Carre%C3%B1o%20F%5bAuthor%5d&cauthor=true&cauthor_uid=22551482), [Blanco Rodríguez I](https://www-ncbi-nlm-nih-gov.proxygsu-uga1.galileo.usg.edu/pubmed/?term=Blanco%20Rodr%C3%ADguez%20I%5bAuthor%5d&cauthor=true&cauthor_uid=22551482), [González Castaño R](https://www-ncbi-nlm-nih-gov.proxygsu-uga1.galileo.usg.edu/pubmed/?term=Gonz%C3%A1lez%20Casta%C3%B1o%20R%5bAuthor%5d&cauthor=true&cauthor_uid=22551482), et al. Control of postoperative pain in knee arthroplasty: single dose femoral nerve block versus continuous femoral block. Rev Esp Anestesiol Reanim. 2012;59(4):204–9. doi:[10.1016/j.redar.2012.02.013](https://doi-org.proxygsu-uga1.galileo.usg.edu/10.1016/j.redar.2012.02.013) PMID:22551482
17. [Singh SI](https://www-ncbi-nlm-nih-gov.ezproxy.shsu.edu/pubmed/?term=Singh%20SI%5BAuthor%5D&cauthor=true&cauthor_uid=23921652), [Rehou S](https://www-ncbi-nlm-nih-gov.ezproxy.shsu.edu/pubmed/?term=Rehou%20S%5BAuthor%5D&cauthor=true&cauthor_uid=23921652), [Marmai KL](https://www-ncbi-nlm-nih-gov.ezproxy.shsu.edu/pubmed/?term=Marmai%20KL%5BAuthor%5D&cauthor=true&cauthor_uid=23921652), [Jones PM](https://www-ncbi-nlm-nih-gov.proxygsu-uga1.galileo.usg.edu/pubmed/?term=Jones%20PM%5bAuthor%5d&cauthor=true&cauthor_uid=23921652). The efficacy of 2 doses of epidural morphine for poscesarean delivery analgesia: a randomized noninferiority trial. [Anesth Analg.](https://www-ncbi-nlm-nih-gov.ezproxy.shsu.edu/pubmed/?term=The+efficacy+of+2+doses+of+epidural+morphine+for+postcesarean+delivery+analgesia%3A+a+randomized+noninferiority+trial.) 2013; 117(3): 677-85. doi: 10.1213/ANE.0b013e31829cfd21 PMID:23921652
18. [Marroquin B](https://www-ncbi-nlm-nih-gov.libproxy.ucl.ac.uk/pubmed/?term=Marroquin%20B%5BAuthor%5D&cauthor=true&cauthor_uid=28185794), [Feng C](https://www-ncbi-nlm-nih-gov.libproxy.ucl.ac.uk/pubmed/?term=Feng%20C%5BAuthor%5D&cauthor=true&cauthor_uid=28185794), [Balofsky A](https://www-ncbi-nlm-nih-gov.libproxy.ucl.ac.uk/pubmed/?term=Balofsky%20A%5BAuthor%5D&cauthor=true&cauthor_uid=28185794), [Edwards K](https://www-ncbi-nlm-nih-gov.proxygsu-uga1.galileo.usg.edu/pubmed/?term=Edwards%20K%5bAuthor%5d&cauthor=true&cauthor_uid=28185794), [Iqbal A](https://www-ncbi-nlm-nih-gov.proxygsu-uga1.galileo.usg.edu/pubmed/?term=Iqbal%20A%5bAuthor%5d&cauthor=true&cauthor_uid=28185794), [Kanel J](https://www-ncbi-nlm-nih-gov.proxygsu-uga1.galileo.usg.edu/pubmed/?term=Kanel%20J%5bAuthor%5d&cauthor=true&cauthor_uid=28185794), et al. Neuraxial opioids for post-cesarean delivery analgesia: can hydromorphone replace morphine? A retrospective study. [Int J Obstet Anesth.](https://www-ncbi-nlm-nih-gov.libproxy.ucl.ac.uk/pubmed/28185794) 2017;30:16-22. doi: 10.1016/j.ijoa.2016.12.008 PMID:28185794
19. Petchara S, Jadesadha T, Supawadee S, [Sakdanuwatwong S](https://www-ncbi-nlm-nih-gov.proxygsu-uga1.galileo.usg.edu/pubmed/?term=Sakdanuwatwong%20S%5bAuthor%5d&cauthor=true&cauthor_uid=27422406), [Piangjai M](https://www-ncbi-nlm-nih-gov.proxygsu-uga1.galileo.usg.edu/pubmed/?term=Piangjai%20M%5bAuthor%5d&cauthor=true&cauthor_uid=27422406). Comparison of continuous femoral nerve block (CFNB/SA) and continuous femoral nerve block with mini-dose spinal morphine (CFNB/SAMO) for postoperative analgesia after total knee arthroplasty (TKA): a randomized controlled study. BMC Anesthesiology. 2016; 16(1):38. doi: 10.1186/s12871-016-0205-2 PMID:27422406

**Supplements**

**Numeric Rating Scale (NRS)**

An 11-point scale where 0 = no pain and 10 = the most severe pain).

**Modified Bromage motor scale**

0 = no motor block, able to lift extended limb off the bed;

1 = partial block, able to flex/extend the knee and ankle;

2 = partial block, only plantar flexion of the ankle possible;

3 = complete block, no voluntary movement of the limb.

**The score of patients’ satisfactions**

1 = poor, 2 = fair, 3 = good, 4 = excellent.

**Definitions of postoperative complications**

| **Complications** | **Requirements for Acceptance** |
| --- | --- |
| Arrhythmia | New onset arrhythmia confirmed by 12-lead electrocardiogram and necessitated medical treatment and/or electroversion |
| Cardiac insufficiency | Requirement of inotropic support for more than 24 hours |
| Acute myocardial infarction | Increase of troponin T concentration above the hospital laboratory’s myocardial infarction threshold and either new Q waves (duration ≥ 0.03 s) or persistent changes (4 days) in ST-T segment |
| Respiratory insufficiency | Requirement of mechanical ventilation for more than 24 hours |
| Pneumonia | New infiltrate on chest x-ray combined with temperature greater than 38°C and leukocytosis |
| Pulmonary embolism | New onset pulmonary embolism confirmed by CTPA |
| Deep vein thrombosis | New onset deep vein thrombosis confirmed by color Doppler ultrasound |
| Stroke | Appearance of persisted new focal neurologic deficit and confirmed by neurologic imaging study |

**12-item short-form health survey (SF-12)**

| 1. In general, would you say your health is: | Excellent | | | Very good | | | | Good | | | | | Fair | | | | | Poor | | |
| --- | --- | --- | --- | --- | --- | --- | --- | --- | --- | --- | --- | --- | --- | --- | --- | --- | --- | --- | --- | --- |
|  | (1) | | | (2) | | | | (3) | | | | | (4) | | | | | (5) | | |
| The following items are about activities you might do during a typical day. Does your health now limit you in these activities? If so, how much? | | | | | | | | | | | | | | | | | | | | |
|  | | | | | | | Yes, Limited A Lot | | | | | Yes, Limited A Little | | | | | No, Not Limited At All | | | |
| 1. Moderate activities, such as moving a table, pushing a vacuum cleaner, bowling, or playing golf | | | | | | | (1) | | | | | (2) | | | | | (3) | | | |
| 1. Climbing several flights of stairs | | | | | | | (1) | | | | | (2) | | | | | (3) | | | |
| During the past 4 weeks, have you had any of the following problems with your work or other regular daily activities as a result of your physical health? | | | | | | | | | | | | | | | | | | | | |
|  | | | | | | | | | | | | | | | YES | | | | | NO |
| 1. Accomplished less than you would like | | | | | | | | | | | | | | | (1) | | | | | (2) |
| 1. Were limited in the kind of work or other activities | | | | | | | | | | | | | | | (1) | | | | | (2) |
| During the past 4 weeks, have you had any of the following problems with your work or other regular daily activities as a result of any emotional problems (such as feeling depressed of anxious)? | | | | | | | | | | | | | | | | | | | | |
|  | | | | | | | | | | | | | | | YES | | | | | NO |
| 1. Accomplished less than you would like | | | | | | | | | | | | | | | (1) | | | | | (2) |
| 1. Didn’t do work or other activities as carefully as usual | | | | | | | | | | | | | | | (1) | | | | | (2) |
| 1. During the past 4 weeks, how much did pain interfere with your normal work (including both work outside the home and housework)? | Not at all | A little bit | | | | Moderately | | | | | Quite a bit | | | | | Extremely | | | | |
|  | (1) | (2) | | | | (3) | | | | | (4) | | | | | (5) | | | | |
| These questions are about how you feel and how things have been with you during the past 4 weeks. For each question, please give the one answer that comes closed to the way you have been feeling. How much of the time during the past 4 weeks. | | | | | | | | | | | | | | | | | | | | |
|  | All of the Time | | Most of the Time | | A Good Bit of the Time | | | | Some of the Time | | | | | A Little of the Times | | | | | None of the Time | |
| 1. Have you felt calm and peaceful? | (1) | | (2) | | (3) | | | | (4) | | | | | (5) | | | | | (6) | |
| 1. Did you have a lot of energy? | (1) | | (2) | | (3) | | | | (4) | | | | | (5) | | | | | (6) | |
| 1. Have you felt dowmhearted and blue? | (1) | | (2) | | (3) | | | | (4) | | | | | (5) | | | | | (6) | |
|  | All of the time | Most of the time | | | | Some of the time | | | | A Little of the time | | | | | | None of the time | | | | |
| 1. During the past 4 weeks, how much of the time has your physical health or emotional problems interfered with your social activities (like visiting with friends, relatives, etc.)? | (1) | (2) | | | | (3) | | | | (4) | | | | | | (5) | | | | |

**Scoring the Medical Outcomes Study SF-12**

There are 3 steps involved in calculating the SF-12 Physical Component Summary (PCS) and Mental Component Summary (MCS) scores.

Step 1. Check for missing or out-of-range values.

Step 2. Convert each item response into both physical and mental standardized values according to the table below.

Step 3. Sum the physical standardized values from step 2 across all 12 items and add 56.57706 to create the SF-12 PCS score. Sum the mental standardized values in similar fashion and add 60.75781 to create the SF-12 MCS score

|  | Item number | Item Response Value | Physical Standardized Value | Mental Standardized Value |
| --- | --- | --- | --- | --- |
| 1 | (General Health) | (1) | 0 | 0 |
|  |  | (2) | -1.31872 | -0.06064 |
|  |  | (3) | -3.02396 | 0.03482 |
|  |  | (4) | -5.56461 | -0.16891 |
|  |  | (5) | -8.37399 | -1.71175 |
| 2 | (Moderate Activities) | (1) | -7.23216 | 3.93115 |
|  |  | (2) | -3.45555 | 1.86840 |
|  |  | (3) | 0 | 0 |
| 3 | (Climbing Several Flights of Stairs) | (1) | -6.24397 | 2.68282 |
|  |  | (2) | -2.73557 | 1.43103 |
|  |  | (3) | 0 | 0 |
| 4 | (Accomplish less than you would like) | (1) | -4.61617 | 1.44060 |
|  |  | (2) | 0 | 0 |
| 5 | (Limited in the kind of activities) | (1) | -5.51747 | 1.66968 |
|  |  | (2) | 0 | 0 |
| 6 | (Accomplish less than you would like) | (1) | 3.04365 | -6.82672 |
|  |  | (2) | 0 | 0 |
| 7 | (Didn’t do activities as carefully as usual) | (1) | 2.32091 | -5.69921 |
|  |  | (2) | 0 | 0 |
| 8 | (Pain interferes with normal work) | (1) | 0 | 0 |
|  |  | (2) | -3.80130 | 0.90384 |
|  |  | (3) | -6.50522 | 1.49384 |
|  |  | (4) | -8.38063 | 1.76691 |
|  |  | (5) | -11.25544 | 1.48619 |
| 9 | (Felt calm and peaceful) | (1) | 0 | 0 |
|  |  | (2) | 0.66514 | -1.94949 |
|  |  | (3) | 1.36689 | -4.09842 |
|  |  | (4) | 2.37241 | -6.31121 |
|  |  | (5) | 2.90426 | -7.92717 |
|  |  | (6) | 3.46638 | -10.19085 |
| 10 | (Have a lot of energy) | (1) | 0 | 0 |
|  |  | (2) | -0.42251 | -0.92057 |
|  |  | (3) | -1.14387 | -1.65178 |
|  |  | (4) | -1.61850 | -3.29805 |
|  |  | (5) | -2.02168 | -4.88962 |
|  |  | (6) | -2.44706 | -6.02409 |
| 11 | (Felt downhearted and blue) | (1) | 4.61446 | -16.15395 |
|  |  | (2) | 3.41593 | -10.77911 |
|  |  | (3) | 2.34247 | -8.09914 |
|  |  | (4) | 1.28044 | -4.59055 |
|  |  | (5) | 0.41188 | -1.95934 |
|  |  | (6) | 0 | 0 |
| 12 | (Health interferes with social activities) | (1) | -0.33682 | -6.29724 |
|  |  | (2) | -0.94342 | -8.26066 |
|  |  | (3) | -0.18043 | -5.63286 |
|  |  | (4) | 0.11038 | -3.13896 |
|  |  | (5) | 0 | 0 |

**WOMAC osteoarthritis index**

| **Pain** | | | | | |
| --- | --- | --- | --- | --- | --- |
|  | None | Slight | Moderate | Severe | Extreme |
| 1. walking | 0 | 1 | 2 | 3 | 4 |
| 1. Stair climbing | 0 | 1 | 2 | 3 | 4 |
| 1. nocturnal | 0 | 1 | 2 | 3 | 4 |
| 1. rest | 0 | 1 | 2 | 3 | 4 |
| 1. weight bering | 0 | 1 | 2 | 3 | 4 |
| **Stiffness:** | | | | | |
|  | None | Slight | Moderate | Severe | Extreme |
| 1. morning stiffness | 0 | 1 | 2 | 3 | 4 |
| 1. stiffness occurring later in the day | 0 | 1 | 2 | 3 | 4 |
| **Physical function:** | | | | | |
|  | None | Slight | Moderate | Severe | Extreme |
| 1. descending stairs | 0 | 1 | 2 | 3 | 4 |
| 1. ascending stairs | 0 | 1 | 2 | 3 | 4 |
| 1. rising from sitting | 0 | 1 | 2 | 3 | 4 |
| 1. standing | 0 | 1 | 2 | 3 | 4 |
| 1. bending to floor | 0 | 1 | 2 | 3 | 4 |
| 1. walking on flat | 0 | 1 | 2 | 3 | 4 |
| 1. getting in or out of car | 0 | 1 | 2 | 3 | 4 |
| 1. going shopping | 0 | 1 | 2 | 3 | 4 |
| 1. putting on socks | 0 | 1 | 2 | 3 | 4 |
| 1. rising from bed | 0 | 1 | 2 | 3 | 4 |
| 1. taking off socks | 0 | 1 | 2 | 3 | 4 |
| 1. lying in bed | 0 | 1 | 2 | 3 | 4 |
| 1. getting into or out of the bathtub | 0 | 1 | 2 | 3 | 4 |
| 1. sitting | 0 | 1 | 2 | 3 | 4 |
| 1. getting on or off toilet | 0 | 1 | 2 | 3 | 4 |
| 1. heavy domestic duties | 0 | 1 | 2 | 3 | 4 |
| 1. light domestic duties | 0 | 1 | 2 | 3 | 4 |
